# Effect of erosive conditions on different sealant materials used in paediatric dentistry

**DOI:** 10.1590/1807-3107bor-2024.vol38.0053

**Published:** 2024-06-24

**Authors:** Jessica Klöckner KNORST, Renan Vaz MACHRY, Ana Carolina CADORE-RODRIGUES, Kiara Serafini DAPIEVE, Daniela HESSE, Clarissa Calil BONIFÁCIO, Thiago Machado ARDENGHI, Cornelis Johannes KLEVERLAAN

**Affiliations:** (a) Universidade Federal de Santa Maria – UFSM, Postgraduate Program in Dental Science, Santa Maria, RS, Brazil.; (b) Academic Centre for Dentistry Amsterdam – ACTA, Department of Paediatric Dentistry, Amsterdam, The Netherlands.; (c) Academic Centre for Dentistry Amsterdam – ACTA, Department of Dental Materials Science, Amsterdam, The Netherlands.

**Keywords:** Pediatric Dentistry, Pit and Fissures Sealants, Child, Oral Health, Sealants

## Abstract

To evaluate the effect of acidic challenge on erosion depth and topographic characteristics of different materials used as occlusal sealants. Two hundred specimens of five sealant materials (Fuji IX, Ketac Molar, Fuji II, Equia and Clinpro) and forty bovine teeth enamel samples (control) were prepared and exposed to acidic challenge. The specimens were immersed in four different solutions (orange juice, coke drink, citric acid or distilled water) under mildly shaken conditions for 3 days. The erosion depth profiles were measured using a profilometer and Scanning Electron Microscope (SEM). Two-way ANOVA with Tukey post-hoc test was performed to evaluate the interactions. Sealant material and acidic challenge had significant effects on erosion depth. Among the materials, Fuji II presented the highest mean of erosion depth after immersion in orange juice, coke drink, and citric acid. All materials groups presented higher erosion depth values after immersion in the citric acid solution, except Clinpro. Bovine enamel presented higher erosion depth values compared to all materials when submitted to erosive challenge. Sealant materials submitted to the acidic challenge presented different degrees of erosion and topographic modification; however, they are less susceptible to erosion than bovine teeth enamel.

## Introduction

The occlusal surface is the most susceptible area for the development of dental caries, as the pit and fissures are difficult to clean and susceptible to the invasion of bacteria since the teeth eruption.^1^ The application of fissure sealants is suggested, since it works as a mechanical barrier facilitating plaque removal by the patient, and preventing the development or progression of caries lesions on theses surfaces.^2^ Therefore, fissures sealants have been reported in the literature as a caries-preventive approach for paediatric patients with poor oral hygiene and with a high risk of dental caries.^1^

Several materials are indicated as occlusal sealants, however, composites or glass ionomer cements (GIC) are more often used.^3^ Among important characteristics of sealants materials, previous studies evaluated the bacterial adhesion to their surface.^4-6^ Evaluating this property is important since colonization by cariogenic bacteria can result in the development of carious lesions in the region adjacent to the sealing material and, therefore, materials with antimicrobial properties are preferred when applied as occlusal sealants.^4-6^ In addition, the difficulty of applying sealants in a humid environment, mainly due to uncooperative children, suggests that GIC can be more easily applied than composites, considering their hydrophilic properties.^7-9^

Factors such as the patient’s diet and the consequent exposure to acid challenge can have an influence on the oral environment.^10^ Thus, since the prevalence and clinical importance of dental erosion have been emphasized in the literature,^11^ it is hypothesized that the dental materials are also subject to the erosive challenge. In this context, topographic changes such as increased surface roughness due to an erosive process may affect the treatment longevity, and especially favour the formation of bacterial colonies on the surface of the restoration,^12^ which can limit the clinical success of the dental materials.

Although some studies have been conducted to assess the effect of acidic challenge on sealant material surface and tooth substrates,^10,13-16^ the data are inconsistent and inconclusive, especially regarding the GIC. In addition, to our best knowledge, the association considering specific occlusal sealants-materials and erosion depth has not been explored yet. Therefore, it is important to know how susceptible these materials are to erosion by acidic beverages often consumed by children and adolescents, especially those with a high-caries risk.^17,18^Therefore, this study evaluated the effect of acidic challenge on erosion depth and topographic characteristics of materials used as occlusal sealants. Our conceptual hypothesis was that acidic solutions have an erosive effect on the surface of sealing materials, being that different acidic solutions promote different degrees of erosion.

## Methodology

### Study design and sample

**Figure 1:**
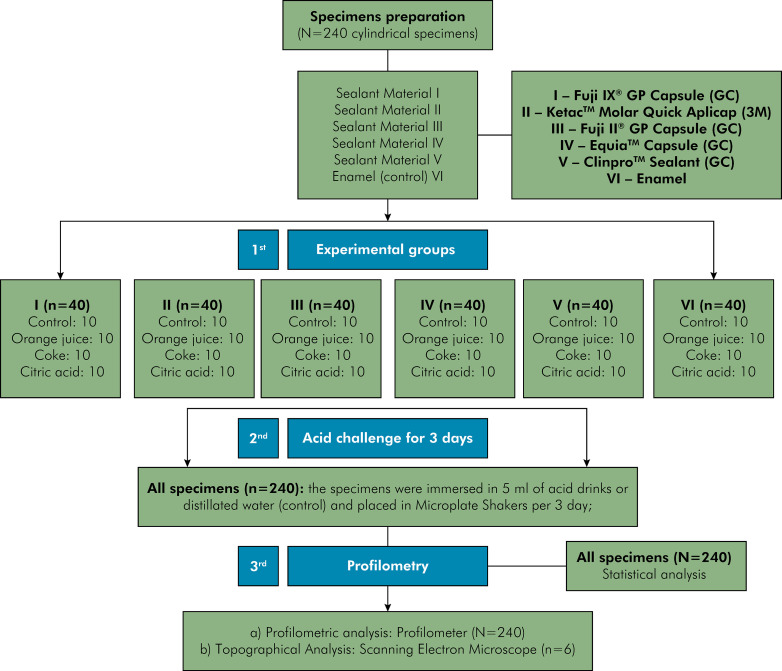
Study design and the general description of the materials used in this study.

The study design and the general description of the materials used in this study are presented in Figure 1 and Table 1. This study was designed with 24 experimental groups (n=10) considering two factors: the “material” factor in six levels: Bovine enamel (control); GIC – three high-viscosity GIC (Fuji IX, Ketac Molar, Equia); low viscosity resin-modified GIC – (Fuji II); RBS – low-viscosity resin-based sealant (Clinpro); and the “acidic challenge” factor in four levels: Ctrl – distilled water (control); OJ – orange juice; CD – cola-based drink; CA – 0.65% citric acid.

**Table 1:** Batch numbers, type of material, and composition of the materials used in this study.

Material (manufacturers) and batch numbers	Type of material	Composition
FUJI XI^®^ (GC Europe, Leuven, Belgium), LOT 210118A	High-viscosity glass-ionomer cement (self-curing)	Alumino-fluoro-silicate glass, polyacrylic acid, distilled water, polybasic carboxylic acid
Ketac™ Molar (3M ESPE, St. Paul, USA), LOT 76577296	High-viscosity glass-ionomer cement	Al-Ca-La fluorosilicate glass, 5% copolymer acid (acrylic and maleic acid), polyalkenoic acid, tartaric acid, water
Equia™ Capsule (GC Europe, Leuven, Belgium), LOT 201222A	High-viscosity glass-ionomer cement (self-curing)	Polyacrylic acid, aluminosilicate glass, distilled water
FUJI II^®^ (GC Europe, Leuven, Belgium), LOT 201117A	Low-viscosity resin-modified glass ionomer cement (light-curing)	Aluminosilicate glass, water, polyacrylic acid, HEMA and UDMA
Clinpro™ Sealant (3M ESPE, St. Paul, USA), LOT 00226146	Low-viscosity resin-based sealant	TEGDMA, BDGDA, tetrabutylammonium, tetrafluoroborate, silane-treated silica

GIC glass-ionomer cement; RM-GIC, resin-modified GIC; RBS, resin-based sealant; FC, Flowable composite; HEMA, 2-hydroxyethyl methacrylate; UDMA, Diurethane dimethacrylate; TEGDMA, triethylene glycol dimethacrylate; BDGDA, bisphenol a diglycidyl ether dimethacrylate.

The sample size was calculated in OpenEpi considering the results of a previous study.^10^ A standard error of 5%, a statistical power of 90%, and a mean difference in the erosive pattern of 0.074 (SD 0.276) and 0.03 (SD 0.101) between resin-based and GIC groups subjected to the acid challenge were considered. The minimum required sample was 6 specimens per group. Considering the possibility of losses due to breakages or failures during the study progress and the sample variability of the bovine teeth used, 10 specimens per group were used.

### Specimen preparation and experimental groups

Forty cylindrical specimens of each sealant material - Fuji IX® GP Capsule (GC Europe, Leuven, Belgium), Ketac™ Molar Quick Aplicap (3M 3M ESPE, St Paul, United States), Fuji II® GP Capsule (GC Europe, Leuven, Belgium), Equia™ Capsule (GC Europe, Leuven, Belgium), Clinpro™ Sealant (3M ESPE, St Paul, United States) were made using cylindrical metal molds (Ø = 10 mm; thickness = 1.0 mm). The molds were placed onto a glass plate and filled with a single layer of each sealant material. A polyester strip was subsequently positioned over the increment and compressed using a glass slide to obtain a flat surface. All materials were manipulated according to the manufactures’ instructions. The self-curing materials (Fuji IX®, Ketac™ Molar Quick Aplicap, Fuji II®, and Equia™, GC Europe, Leuven, Belgium) were kept in room temperature (24ºC) until the setting was completely concluded. For light-curing material (Clinpro™), each specimen was light-cured using a high intensity light instrument (Elipar FreeLight 2; 3M ESPE, St Paul, United States) for 20 seconds. To obtain the enamel specimens, freshly extracted bovine incisors were sectioned at the cement–enamel junction using a diamond disk (Isomet 1000; Buehler, Lake Bluff, Plymouth, USA) to separate the crowns from the roots. Cylindrically shaped specimens (Ø = 10 mm) were prepared perpendicular to the labial surface with a hollow water-cooled diamond drill. All specimens (sealant materials and enamel) were polished with silicon carbide papers (600- and 1200-grit) (Buehler, Lake Bluff, USA) under water-cooling in a polishing machine, thoroughly washed with distilled water, and dried. All the specimens were stored in distilled water (12 days up to 18 days; 37ºC) prior the acidic challenge.

### Acidic challenge

**Figure 2:**
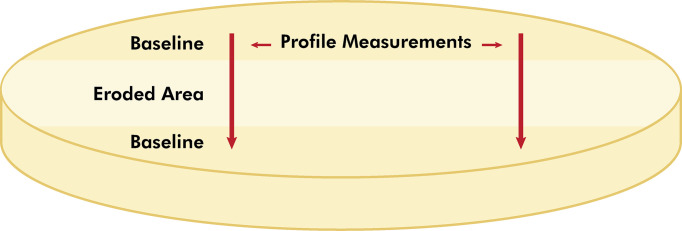
Specimen model used to profilometric measurements of material loss.

The polished surface of all specimens was partly covered with adhesive tape (Tesa; Beiersdorf, Hamburg, Germany). The strips were applied to the right and left sides of the surface, leaving a treatment window of unprotected sealant material/enamel of 1.5 mm x 10 mm (Figure 2). All 240 specimens (n = 40) were randomly distributed in four groups according to the solutions used (n = 10). In the experimental groups, the samples were immersed in different solutions being each specimen placed in a test tube containing 5.0 mL of the orange juice (pH 4.08), cola-based drink (pH 2.75), or citric acid 0.65% (pH 3.60). The pH of the solutions was measured with a pH meter (Mettler Delta 350, Mettler Toledo, Leicester, United Kingdom) before the acidic challenge to ensure standardization of solutions in all materials evaluated. In the Ctrl group, the specimens were immersed in 5.0 ml of distilled water. The immersed specimens were subjected under mildly shaken (100 rpm) conditions for 3 days at room temperature (23ºC).

### Profilometry

**Figure 3:**
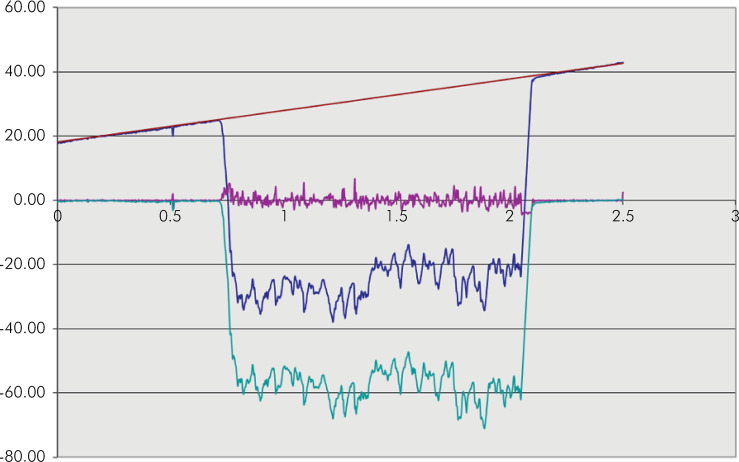
Profilometric profile model obtained to assess the erosion depth.

After acid challenge was completed, the adhesive tapes were removed and the disc specimens were cleaned in an ultrasonic bath (Vitasonic, Vita Zanhfabrik; Bad Sackingen, Germany) with distilled water (≅ 40 mL, 5 min). Two profiles were obtained (Mitutoyo SJ 400 Profilometer, Mitutoyo Corporation, Kawasaki, Japan) at the centre of the specimens with a measuring speed of 0.5 mm/s and a cut-off of 2.5 mm. The tape-covered zone served as the reference area (baseline) for profilometric measurements of material loss (Figure 2). The distance between the two profiles was approximately 5 mm. The average depth of the eroded part of the specimen was calculated using dedicated software. A baseline correction on the two reference planes was carried out. On the baseline-corrected profile, the width of the eroded part was determined at the steepest points of the profile. The mean erosion depth was determined by measuring the mean depth between the two baseline areas (Figure 2 and 3). The results of two measurements were averaged for each sample and the material loss of a given sample was expressed as the mean of two scans.

### Qualitative topographic analysis

Additional specimens (n = 1) for each sealant material and enamel were inspected regarding the topographical changes after acidic challenge, and citric acid was selected as the standard solution for this analysis. The samples were cleaned in an ultrasonic bath (Vitasonic, Vita Zanhfabrik; Bad Sackingen, Germany) with distilled water (≅40 mL, 5 min). Then the specimens were gold-sputtered (Edwards S150B, BOC Edwards, Burgess Hill, United Kingdom) and analyzed under Scanning Electron Microscopy (SEM, Evo LS15, Oberkochen, Carl Zeiss, SEM, Evo LS15, Carl Zeiss, Oberkochen, Germany) at 100, 500 and 10k× magnification.

### Data analysis

Profilometry data were analysed using the STATA 16.0 program (Stata Corporation, College Station, United States). A descriptive analysis of mean erosion depth according to each material group and test solution was performed. Two-way ANOVA was performed to identify the interaction between the independent (material, solution) and dependent (erosion depth) variables, followed by the Tukey post-hoc test for the interaction (material × solution). A significance level of 0.05 was considered.

## Results

According to the two-way ANOVA, the material (p < 0.001, F = 89.01) and acidic challenge (p < 0.001, F = 210.99) had statistically significant effects. In addition, a statistically significant interaction was found between the sealant material x acidic challenge (p < 0.001, F = 29.16).

Enamel specimens (Ctrl) exhibited the most significant depth of erosion following acidic challenge (p < 0.01), with citric acid solution demonstrating the highest erosive effect (78.13 [SD] 26.06). Among the sealant materials, Fuji II displayed the highest mean erosion depths in orange juice (3.98 [2.55]), coke drink (6.34 [4.68]), and citric acid solution (51.60 [8.01]), albeit without statistical significance for orange juice and coke drink. Notably, all material groups showed significant erosion in citric acid solution, except for Clinpro (Table 2).

**Table 2:** Mean and standard deviation of erosion depth (in µm) of sealant materials and enamel after 3 days of exposure to different solutions, obtained by the profilometry analysis (n = 240).

Material	Acidic challenge			

	Water (Ctrl)	Orange juice	Coke drink	Citric acid
Enamel (Ctrl)	0.23 (0.18) ^Ac^	26.86 (7.54) ^Ab^	18.53 (19.04) ^Ab^	78.13 (26.06) ^Aa^
Fuji IX	2.51 (2.29) ^Ab^	1.86 (1.13) ^Bb^	4.76 (1.91) ^Bb^	32.02 (5.83) ^Ca^
Ketac Molar	1.93 (0.98) ^Ab^	1.81 (1.14) ^Bb^	2.12 (3.36) ^Bb^	15.24 (6.81) ^Da^
Equia	1.15 (0.47) ^Ab^	0.55 (0.35) ^Bb^	0.87 (1.23) ^Bb^	13.39 (4.17) ^Ea^
Fuji II	3.15 (2.26) ^Ab^	3.98 (2.55) ^Bb^	6.34 (4.68) ^A,Bb^	51.60 (8.01) ^Ba^
Clinpro	0.32 (0.41) ^Aa^	0.85 (0.58) ^Ba^	0.65 (0.48) ^Ba^	1.06 (0.56) ^Fa^

Different upper-case letters: statistical difference (p<0.05) considering the material factor (column); Different lower-case letters: statistical difference (p<0.05) considering the solution factor (lines).

The Fuji IX, Ketac Molar, and Equia groups exhibited intermediate erosion values in response to the Citric Acid solution, with statistically significant differences among them (p < 0.05; Fuji IX > Ketac Molar > Equia). However, for the other solutions, similar results were obtained without statistical differences (p > 0.05).

In Table 3, SEM topographic images illustrating depth erosion after acidic challenge for each sealant material are presented. At a lower magnification (100×), the baseline areas (covered with tape during immersion in the solutions) are visible at the top and bottom of the image. The eroded area, situated between the baseline areas, is evident in all groups, except for Clinpro, which showed no detectable changes under microscopy. Intermediate magnification (500×) images highlight the border between the baseline and eroded areas. Finally, at higher magnification (10k×), both baseline and eroded areas are distinctly represented.

**Table 3:** Representative topographic images in scanning electron microscopy (SEM; 100×, 500× and 10K×) of the specimens after the acid citric challenge.

Group	Topographic images			

	SEM (100×)	SEM (500×)	SEM (10K×)	
			Baseline	Eroded area
Enamel (Ctrl)	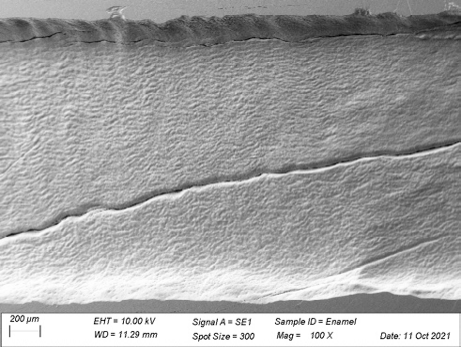	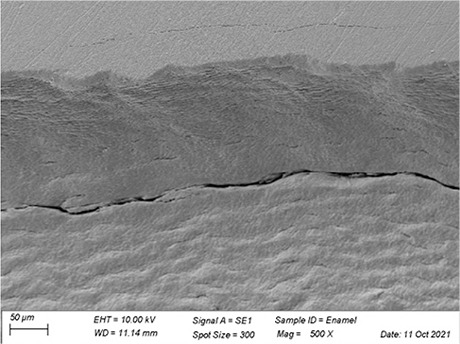	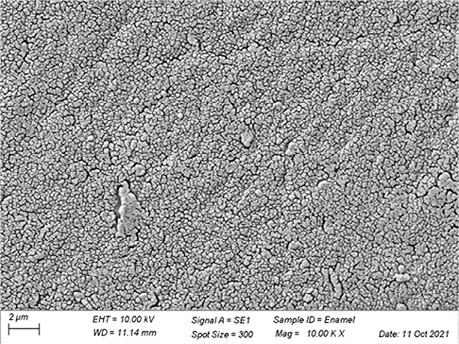	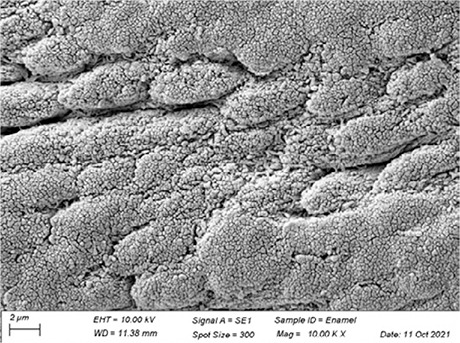
Fuji IX	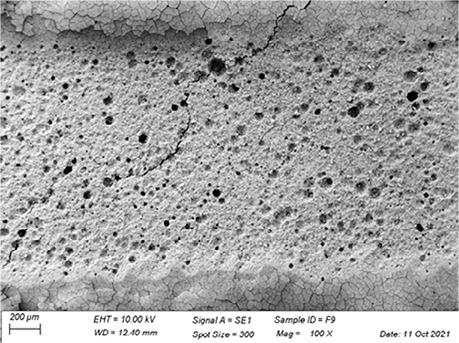	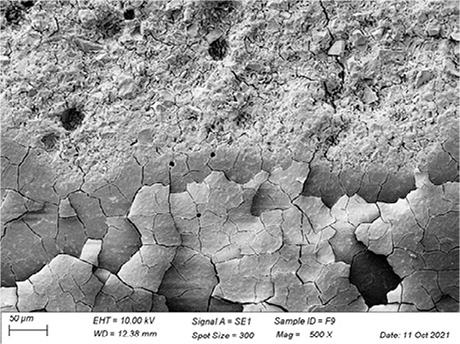	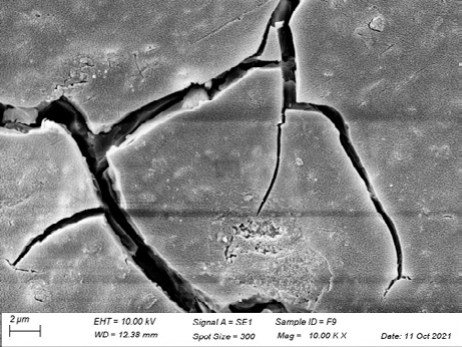	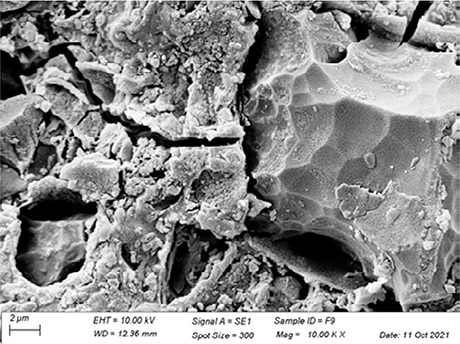

## Discussion

This study evaluated the erosive profile of materials used as occlusal sealants submitted to acidic challenge in different acidic solutions. Overall, the results for erosion depth data showed that the material and acidic challenge had statistically significant effects. In addition, a statistically significant interaction was found between the sealant material x acidic challenge.

Considering the sealant materials, the resin-modified low-viscosity GIC (Fuji II) presented the highest erosion depth in all solutions, but with statistic difference only for citric acid. A previous study also showed higher erosion values for the resin-modified low-viscosity GIC,^19,20^ which may be related to the composition of these cements. The GIC materials are based on the reaction of a silicate glass-powder and a mixture of various polyalkenoate acids. Low-viscosity GICs are used as sealants due to their lower tension and good fluidity, which results in greater infiltration into pits and fissures. On the other hand, since the amount of water inside the cement matrix is higher in these materials when compared to the resin-based materials, it results in worse mechanical properties, including higher susceptibility to erosion and wear.^19,21^

Our findings suggested that all materials used in this study, in general, presented lower erosion depth values than bovine tooth enamel subject to acidic challenge. Enamel is a highly mineralized tissue formed by calcium phosphate and hydroxyapatite crystals, which are soluble in an aqueous environment.^22^ The integrity of the tooth’s hydroxyapatite is ruled by the pH of the oral fluids,^22^. Considering carious context, the pH becomes critical when oral fluids are saturated in relation to the hydroxyapatite of the tooth. Above this value, no enamel demineralization process develops. Taking as a parameter the value of 5.5 as critical pH for human enamel,^23^ all acidic substances with a pH below 5.5 can dissolve the hydroxyapatite crystals. The solutions employed in our study all had a pH lower than 5.5 (orange juice pH 4.08, cola-based drink pH 2.75, or citric acid 0.65% pH 3.60), which likely accounts for our findings. It’s essential to note that our investigation doesn’t pertain to the process of demineralization due to caries; rather, it focuses on the impact of ingesting acidic drinks. While pH is a crucial factor in assessing the erosive potential of a beverage or food, it’s not the sole determinant^24^. Many lower pH solutions may not dissolve enamel due to their high concentrations of Calcium (Ca) and Phosphate (P), essential mineral ions for dental minerals. These ions can lead to saturation or even supersaturation, preventing demineralization^24^. Therefore, it is noteworthy that the development of dental erosion depends on the concentrations of calcium and phosphate ions in saliva and the availability of fluoride to act in the remineralization process,^25^ which was not evaluated in our study. Future studies could explore beverages such as yogurt, which, despite its low pH, contains significant amounts of Ca, offering a different perspective on erosive potential.

Acid solutions are part of the diet of children and adolescents and, therefore, restorative materials applied in the oral cavity of these individuals are naturally submitted to topographical changes due to the acidic challenges.^10,13-16^ In our study, the five materials evaluated did not show statistically significant erosion depth values when subjected to acidic beverages commonly consumed by children. However, a citric acid solution was also used to simulate these acidic beverages in a more controlled way,^26^ excluding possible confounding components that may be present in other beverages^27^. Only the resin-based sealant (Clinpro) was not negatively affected by exposure to this acidic solution. Previous studies have consistently demonstrated that resin-based sealants have a higher retention rate and longevity than glass-based sealants,^28^ as well as less erosive wear.^10^ A possible explanation for the greater stability of composites may be due to the formulation of the material and the morphology of the filler particles, which are nanosized and regular, allowing the incorporation of a large inorganic volume.^29,30^ However, as occlusal sealants are primarily indicated to prevent dental caries, controversial results are also demonstrated, pointing to the inexistence of difference between the two types of materials, especially regarding the prevention of dental caries.^5^

Our SEM images corroborate the data obtained in the profilometry analysis. At lower magnification (100×) the topographic differences between the reference areas and the eroded area are evident, with the exception of the Clinpro group, which did not show detectable changes neither under microscopy, nor in the profilometry analysis. In this sense, an observation is made to the Equia group, which showed no quantitative change identified in the profilometry, but did show visible qualitative topographic changes in SEM images. The images presented a porous surface, indicating not only the loss of material, but also an increase in the surface roughness, suggesting a greater susceptibility to microbial adhesion.^12^ To support this hypothesis, specific analyses are needed in order to assess bacterial adhesion to these materials and therefore further studies are suggested.

This study has some limitations that should be acknowledged, such as the acidic challenge period. Our specimens were submitted to acidic challenge for three days, which may be considered a short period of time, since previous studies used seven days.^10^ However, long-term protocols of acid challenge may lead to overexposure of the samples to solutions that are usually consumed at sporadic times in the daily diet of children or adolescents. In our study, expressive values of erosion depth were observed in all evaluated solutions after three days of acid challenge; therefore, we consider this a clinically significant parameter. In addition, it could be suggested that future studies use demineralization-remineralization protocols for a broader view of the erosive process. However, although this additional process can influence the data obtained for tooth enamel, it has no impact on the results of the sealants materials evaluated. Another possible limitation of our study is the fact that bovine teeth were used instead of human teeth. However, a recent systematic review demonstrated that both human and bovine teeth behave similarly in studies investigating dental adhesion in enamel and dentin,^31^and the use of bovine teeth has been encouraged.

Finally, dietary control in preventive treatments for children and adolescents remains essential to avoid structural losses due to erosion of the enamel and sealant materials, maintaining structural balance and topography. This knowledge indicates that the materials are deemed suitable for utilization in the evaluated context, with the goal of enhancing treatment success across diverse social and behavioral contexts, particularly for children at high risk of caries. It is imperative, however, to emphasize that the applicability of these recommendations within the clinical setting necessitates further validation through clinical studies.

Our findings showed that bovine enamel specimens exhibited higher erosion depth compared to the different sealant materials, with higher erosion levels presented in the citric acid solution. Among the sealant materials, the low viscosity resin-modified GIC presented the highest means of erosion depth, while the resin-based sealant showed the best performance.
